# From a Hetero- to a Methylotrophic Lifestyle: Flash Back on the Engineering Strategies to Create Synthetic Methanol-User Strains

**DOI:** 10.3389/fbioe.2022.907861

**Published:** 2022-06-08

**Authors:** Camille Peiro, Cláudia M. Vicente, Denis Jallet, Stephanie Heux

**Affiliations:** TBI, Université de Toulouse, CNRS, INRAE, INSA, Toulouse, France

**Keywords:** synthetic methylotrophs, methanol, metabolic engineering, modelling, biotechnology

## Abstract

Engineering microorganisms to grow on alternative feedstocks is crucial not just because of the indisputable biotechnological applications but also to deepen our understanding of microbial metabolism. One-carbon (C1) substrate metabolism has been the focus of extensive research for the prominent role of C1 compounds in establishing a circular bioeconomy. Methanol in particular holds great promise as it can be produced directly from greenhouse gases methane and carbon dioxide using renewable resources. Synthetic methylotrophy, i.e. introducing a non-native methanol utilization pathway into a model host, has therefore been the focus of long-time efforts and is perhaps the pinnacle of metabolic engineering. It entails completely changing a microorganism’s lifestyle, from breaking up multi-carbon nutrients for growth to building C-C bonds from a single-carbon molecule to obtain all metabolites necessary to biomass formation as well as energy. The frontiers of synthetic methylotrophy have been pushed further than ever before and in this review, we outline the advances that paved the way for the more recent accomplishments. These include optimizing the host’s metabolism, “copy and pasting” naturally existing methylotrophic pathways, “mixing and matching” enzymes to build new pathways, and even creating novel enzymatic functions to obtain strains that are able to grow solely on methanol. Finally, new approaches are contemplated to further advance the field and succeed in obtaining a strain that efficiently grows on methanol and allows C1-based production of added-value compounds.

## Introduction

Synthetic methylotrophy refers to the design and engineering of methanol assimilation pathways into established non-methylotrophic production hosts making use of their vast biotechnological potential (Becker et al., 2015) and providing access to methanol as feedstock.

To date, attempts to introduce methylotrophy into biotechnologically relevant microbes have been described for *Escherichia coli* (Muller et al., 2015; Whitaker et al., 2017), *Corynebacterium glutamicum* (Lessmeier et al., 2015; Witthoff et al., 2015), *Pseudomonas putida* (Koopman et al., 2009), *Saccharomyces cerevisiae* (Dai et al., 2017) and *Yarrowia lipolytica* (Vartiainen et al., 2019). Efforts to engineer this non-native nutrient catabolism have relied on four different levels of engineering (Erb et al., 2017). In the first level, existing pathways are used to allow methanol assimilation in the non-methylotrophic host (Espinosa et al., 2020). In the next level, synthetic methylotrophs were obtained by “copying and pasting” naturally occurring methanol assimilation pathways (Muller, et al., 2015; Dai, et al., 2017). In the third level, synthetic methanol assimilation pathways were developed by “mixing and matching” enzymes from these methanol assimilation pathways (De Simone et al., 2020). In the last level, novel methanol assimilation pathways were created from known or new enzyme mechanisms (Siegel et al., 2015). However, none of these synthetic strains was able to grow on methanol alone.

If the implementation of methylotrophy in non-native methylotrophs can be seen as a quite straightforward approach, researchers quickly realised that the complexity of methylotrophy could not be reduced to a simple metabolic transplant. In contrast with heterotrophic metabolism where multi-carbon substrates are essentially broken down to obtain metabolites and biomass building blocks, in methylotrophy all carbon-carbon bonds essential to life must be built from a single carbon (C1) molecule. Furthermore, in all these implemented pathways carbon is assimilated in the form of formaldehyde, a central but highly toxic intermediate that can lead to cell death in case of imbalance between dissimilation and assimilation. In the engineering strategies based on naturally occurring methanol assimilation pathways, formaldehyde assimilation is achieved by a cyclic process and requires a C1-acceptor that enables the formation of a C-C bond (Wendisch et al., 2021). The efficiency of methanol assimilation is then determined by the capability of the cells to produce and regenerate the C1-acceptor, especially when C1 is assimilated as formaldehyde. In contrast, the novel pathways, created from novel reactions, are linear and thus independent from any C1-acceptor. However, as formaldehyde is the only intermediate, a high concentration of formaldehyde is required to support a sustainable rate of formaldehyde condensation that enables the formation of a C-C bond. The efficiency of methanol assimilation is then determined by the capability of the cells to tolerate a higher level of formaldehyde. To overcome these obstacles, different rational and evolutionary engineering strategies have been applied during the last decade. The ultimate growth phenotype was recently achieved by Chen et al., 2020 and Kim et al., 2020 who observed growth on methanol as the sole carbon source by a synthetic methylotrophic *E. coli* (Chen et al., 2020; Kim et al., 2020). This review proposes to present the various strategies implemented to reprogram the food diet of non-methylotrophic production hosts.

## Existing Pathways

In this approach, existing pathways within the natural host are engineered (i.e. through gene deletion or overexpression) to improve their overall capacity towards a given function. A few studies have followed this approach to implement synthetic methylotrophy.

In *S. cerevisiae*, when grown with methanol (plus yeast extract), the final optical density increased by 39% relative to medium without methanol. In addition, ^13^C from labeled methanol was incorporated into intracellular metabolites (60% of the acetyl-coA pool was fully labeled). Based on these observations, Espinosa et al. decided to further boost the assimilation of methanol in *S. cerevisiae* by adaptive laboratory evolution (ALE). Genomic analysis of the resulting evolved strains helped in the identification of beneficial mutations allowing improvement of methanol utilization. These mutations were implemented in a wild type strain and the reconstructed strain exhibited a higher biomass yield on methanol (a 21% increase in OD600) than the parental strain. In addition, a ^13^C-methanol tracer analysis revealed that the reconstructed strain had a higher percentage of fully ^13^C-labelled intracellular metabolites (33% of fructose-1,6-bisphosphate and 60% of acetyl-CoA fully labelled) compared with the parental strain (Espinosa, et al., 2020).

It is interesting to notice that other non-methylotrophic microorganisms possess endogenous enzymes that may confer a native capacity for methylotrophy. Endogenous methanol oxidizing activities, which may stem from promiscuous alcohol dehydrogenases, have been observed in Y. *lipolitica* (Koopman, et al., 2009) and *P. putida* (Vartiainen, et al., 2019). *Bacillus subtilis* possesses a Ribulose Monophosphate (RuMP) pathway which may act as a detoxification system for formaldehyde (Yasueda et al., 1999). However, no studies report the use of ALE to optimize the native methylotrophic metabolism of those microorganisms.

## Copy and Paste Enzymes

In this more advanced approach, naturally occurring metabolic pathways for methanol assimilation are implemented in another host and optimized by shaping the metabolic network of the host to fit the acquired property. Aerobic natural methylotrophy is supported by four different metabolic pathways found among methylotrophic bacteria and yeast: the RuMP pathway, the Xylulose Monophosphate (XuMP) pathway, the Calvin-Benson-Bassham (CBB) cycle and the Serine cycle (Wendisch, et al., 2021). In thoses studies aiming at “copying and pasting” existing methylotrophic pathways into non-methylotrophic hosts, the RuMP pathway has been used far more frequently than the others.

### The RuMP Pathway and Its Optimization

In this pathway only three heterologous enzymes, i.e. a methanol dehydrogenase (Mdh), a 3-hexulose-6-phophate synthase (Hps) and a 6-phospho-3-hexuloisomerase (Phi) are needed to integrate the methylotrophic module (Yurimoto et al., 2009). The challenging parts often consist in connecting methanol assimilation to the host central metabolism and in ensuring proper C1-acceptor regeneration.

#### Rational Engineering

##### Screening and Engineering of Mdh

The oxidation of methanol to formaldehyde, the first step in methanol assimilation, is ensured by an Mdh. Depending on the electron acceptor, Mdh enzymes can be classified into three groups: the periplasmic pyrrolo-quinoline-quinone (PQQ)-dependent Mdh found in Gram-negative methylotrophs, the cytoplasmic NAD-dependent Mdh common in Gram-positive methylotrophs and the cytoplasmic NDMA (N,N-dimethyl-4-nitrosoaniline)-dependent Mdh found in *Mycobacterium* and that uses mycofactocin as an *in vivo* electron acceptor (Heux et al., 2018; Dubey et al., 2019). The PQQ- and NDMA-dependent Mdhs are often multimeric proteins (Keltjens et al., 2014; Dubey, et al., 2019) and require the synthesis of PQQ and mycofactocin through complex biosynthetic pathways absent in a lot of hosts. The PQQ-dependent Mdh requires oxygen and specific cellular locations for its proper function while the NAD-dependent Mdh is located in the cytoplasm and functions under both aerobic and anaerobic conditions. For all these reasons, the choice of a NAD-dependent Mdh appeared to be the simplest way to implement synthetic methylotrophy. The *mdh* gene from *Bacillus methanolicus* was the first to be used (Lessmeier, et al., 2015; Muller, et al., 2015; Witthoff, et al., 2015). However, its efficiency towards methanol conversion remains relatively low and the same states for several reported variants. Thereby it required a high amount of methanol to yield high amounts of intracellular labelling (25% ^13^C-enrichment in phosphoenolpyruvate (PEP) with 1M of ^13^C-methanol) (Muller, et al., 2015). Scientists therefore aimed to find new NAD-dependent Mdhs with better kinetic parameters by screening Mdhs coming from different donor organisms. Among them, an alcohol dehydrogenase from the non-methylotrophic *Bacillus stearothermophilus* was found to use methanol as a substrate with a 10 times lower *K*_m_ value (Sheehan et al., 1988). Whitaker and colleagues used this Mdh combined with Hps and Phi from *B. methanolicus* in *E. coli* and showed that the combination resulted in a 43% increase in biomass yield and a 10 times higher ^13^C-enrichment in intracellular metabolites compared to the association using the Mdh from *B. methanolicus* in a media containing 16 times less methanol (i.e. 60 mM vs 1M) but yeast extract (Whitaker, et al., 2017). Another alcohol dehydrogenase from *Cupriavidus necator* was found to exhibit similar activity towards methanol at 30°C to the Mdh from *B. methanolicus* at 45°C (its optimal temperature for activity). To further improve the kinetic parameters of Mdh, engineering strategies were applied. By using directed molecular evolution, a variant of the Mdh from *C. necator* with a catalytic efficiency for methanol 6-fold higher than the wild-type was obtained (Wu et al., 2016). In another study, by using phage assisted evolution on the Mdh2 of *B. methanolicus*, a two times higher methanol incorporation was observed in the resulting strain compared to the strain expressing the native Mdh2 (Roth et al., 2019).

##### Engineering of the Cell Redox State

Cellular redox state directly affects the Mdh activity since high NADH/NAD^+^ ratios are unfavourable to methanol oxidation. Results obtained after ALE experiments aiming at improving methanol assimilation in *E*. *coli* showed mutations in the *nadR* gene encoding for a transcriptional regulator of genes involved in NAD^+^ transport and *de novo* synthesis (Meyer et al., 2018). The activity of this repressor was found to be reduced, highlighting the importance of the NADH/NAD^+^ ratio during growth on methanol. To balance the redox state of the cell, authors also showed that when the NAD-dependent malate dehydrogenase gene *maldh* was knocked-out, growth was improved on methanol and gluconate. This was also observed in *E. coli* during growth on methanol and yeast extract even if methanol assimilation was not improved (Rohlhill et al., 2020). In a similar way, the methanol oxidation rate was improved when Mdh was coupled with a “NADH sink” by using lactate dehydrogenase to recycle NADH into NAD^+^ (Price et al., 2016).

##### Screening of Hps and Phi Candidates

The implementation of a methylotrophic pathway in *E. coli* was done by testing a series of Hps and Phi candidates from different donor organisms *in vitro* and *in vivo* (Muller, et al., 2015; Whitaker, et al., 2017; Fan et al., 2018). In all these studies, Hps and Phi from *B. methanolicus* were the best performing enzymes both *in vitro* and *in vivo*. In *C. glutamicum*, methanol utilization has been achieved by expressing Mdh from *B. methanolicus* together with *hxlA* (3-hexulose-phosphate synthase) and *hxlB* (6-phospho-3-hexuloisomerase) from *B. subtilis* (Lessmeier, et al., 2015; Witthoff, et al., 2015)*.* In the resulting strains, the incorporation of ^13^C-label from ^13^C-methanol into central metabolites was detected, demonstrating the *in vivo* operation of the synthetic methanol utilization pathway (Lessmeier, et al., 2015; Witthoff, et al., 2015). However, expression of the same genes in *S. cerevisiae* failed to allow methanol consumption and cell growth in a minimal medium containing methanol as the sole carbon source (Dai, et al., 2017). In the bacterium *P. putida*, introduction of the *hps* and *phi* genes from *Bacillus brevis* allowed the strain to utilize methanol and formaldehyde as auxiliary substrates (Koopman, et al., 2009). The Hps and Phi expressing strain showed a two times higher biomass yield compared to the control strain when grown on a medium containing formaldehyde plus glucose. Furthermore, the strain was also able to grow when replacing formaldehyde by methanol while the control strain did not reach steady state under these conditions. However, authors did not show any evidence that a functional RuMP was operating *in vivo*. Overall, these studies demonstrate that the efficiency of Hps and Phi is not only linked with their origin but also with the host in which they are expressed. Improvement of Hps and Phi catalytic efficiency has been obtained by fusing both enzymes together and this will be discussed in the section Spatial engineering.

##### Engineering of the Dissimilatory and Recycling Pathways

In order to drive the metabolism towards methanol assimilation, a common strategy is to delete one or more genes encoding for the formaldehyde detoxification pathway to avoid a carbon loss as CO_2_ (Whitaker, et al., 2017; Bennett et al., 2018; Vartiainen, et al., 2019; De Simone, et al., 2020; Rohlhill, et al., 2020). The importance of this mutation has been confirmed in ALE experiments where all the evolved strains with improved methylotrophic capacity had a deletion in one or more genes of the formaldehyde detoxification operon (Chen et al., 2018; Meyer, et al., 2018; Chen, et al., 2020).

Another strategy to stimulate methanol assimilation was to favour the recycling of the C1-acceptor (i.e. Ribulose-5-phosphate, Ru5P). In synthetic methylotrophs, Ru5P regeneration is ensured by the non-oxidative part of the pentose phosphate pathway (PPP). In the natural methylotroph *B. methanolicus*, it was shown that *pfk*, *rpe*, *tkt*, *glpX*, and *fba* (i.e. part of the sedoheptulose-1,7-biphosphatase (SBPase) variant of the RuMP pathway) are key genes involved in Ru5P regeneration. Their deletion resulted in the loss of the capacity of the bacteria to grow on methanol (Brautaset et al., 2004). Therefore, enhancing the host’s capacity to regenerate Ru5P by overexpressing heterologous enzymes appears critical to achieve efficient synthetic methylotrophy. To boost Ru5P regeneration, Bennett et al. overexpressed PPP enzymes by integrating *pfk*, *rpe*, *tkt*, *glpX* and *fba* from *B. methanolicus* under a strong inducible promoter into the *E. coli* chromosome (Bennett, et al., 2018)*.* Even if culture media had to be supplemented with yeast extract, the resulting strain achieved a 20% improvement in biomass production during growth on methanol compared to the parental strain and 59% of ^13^C-enrichment was reached in PEP*.* PEP is an interesting metabolite to follow methanol assimilation and Ru5P recycling. Indeed, the more carbons in PEP are labelled, the more methanol has been assimilated and so the more Ru5P has been recycled. Overexpressing the PPP enzymes does not suffice, as a fine balance must be found between the amounts of methanol carbon directed towards recycling vs biomass formation. Woolston et al. used iodoacetate to block glyceraldehyde-3-phosphate dehydrogenase (Ga3PDH). Authors hypothesised that by inhibiting this reaction, which connects the RuMP pathway to lower glycolysis, larger intermediate pools of the upper glycolytic and RuMP pathways could be maintained in starved cells. This strategy led to a higher methanol incorporation into central metabolites in *E. coli.* The ^13^C-enrichment in fructose-6-phosphate (F6P) reached 27.5% when iodoacetate was present. Moreover, the intracellular concentrations of F6P, Ru5P and sedoheptulose-7-phosphate (S7P) were higher (Woolston et al., 2018). Rohlhill et al. went one step further by modulating the expression of *rpe* and *tkt* using the native formaldehyde-inducible promoter P_*frm*_ of *E. coli*. When combined with the disruption of the malate dehydrogenase, methanol carbon incorporation into intracellular metabolites was improved, but yeast extract was still needed during the experiment (Rohlhill, et al., 2020).

To boost both assimilation and recycling, Chen et al. first integrated two operons in *E. coli*’s genome. The first one included *mdh*, *hps* and *phi* genes while the second one was composed by the same *mdh* and *phi* genes plus the *hps, tkt* and *tal* genes from various organisms. Adding the *tkt* and *tal* genes in the operon helped enhancing Ru5P recycling (Chen, et al., 2020). Moreover, after the first ALE experiment, using Ensemble Modelling for Robustness Analysis (EMRA), authors identified that the high activity of phosphofructokinase and Ga3PDH was channelling the flux away from the RuMP cycle, which tended to unbalance the metabolic system. To reduce the activity of these two enzymes, *pfkA* was knockout and the *gapA* gene was replaced by another *gapC* from *E. coli* BL21 encoding for a less efficient Ga3PDH than the native one.

##### Engineering of the Regulation of Gene Expression

Natural methylotrophs have developed complex regulatory networks allowing them to express the genes required for the methanol metabolism via sensing methanol and/or formaldehyde (Jakobsen et al., 2006). In contrast, synthetic methylotrophs do not possess such genetic machinery to recognize methanol. Therefore, attempts to make synthetic methylotrophs sensing methanol have been carried out. A chimeric two-component system was created in *E*. *coli*, MxaYZ, by fusing the periplasmic methanol sensing domain MxaY of the Mdh from *Paracoccus denitrificans* with the cytoplasmic catalytic transmitter domain of EnvZ from *E. coli* (Ganesh et al., 2017). MxaYZ sensed extracellular methanol and could activate the expression of a fluorescent reporter. Formaldehyde on the other hand is naturally encountered by *E*. *coli* via the formaldehyde-inducible promoter (P_*frm*_) which activates the dissimilatory pathway to avoid any intracellular formaldehyde accumulation. Several examples of engineered P_*frm*_ in synthetic methylotrophs have been described (Denby et al., 2016; Rohlhill et al., 2017). In *E. coli,* P_*frm*_ is situated upstream to the *frmRAB* operon and is repressed by FrmR, a transcriptional repressor. When formaldehyde is present in the cells, it interacts with FrmR and triggers conformational changes, leading to the dissociation of FrmR from its recognized DNA motif within P_*frm*_ and to the derepression of the transcription of the operon (Denby, et al., 2016). When placed upstream the *mdh*, *hps* and *phi* operon, both the native and engineered P_*frm*_ enabled improved biomass production in the engineered strain (Rohlhill, et al., 2017). In another study, FrmR and P_*frm*_ were used to build a formaldehyde biosensor in *E. coli* and to modulate the expression of *mdh*, *hps* and *phi* (Woolston, et al., 2018).

#### Evolutionary Engineering

Despite these efforts, rational engineering strategies were not sufficient to ensure full synthetic C1-assimilation in microorganisms. Rational engineering had to be associated with evolutionary engineering to improve the microorganism’s performance. However, as no growth on pure methanol was initially observed in the synthetic methylotrophs, the strategy adopted by several groups was to first build an “auxotrophic” strain in which methanol is necessary to ensure growth when another carbon source (i.e. glucose, xylose, ribose, gluconate or pyruvate) is present and then to subject the strain to ALE (Chen, et al., 2018; Meyer, et al., 2018; Bennett et al., 2020b; Chen, et al., 2020; Keller et al., 2020).

##### Chassis Optimization to Engineer Methanol-dependent Strain for Growth

**FIGURE 1:**
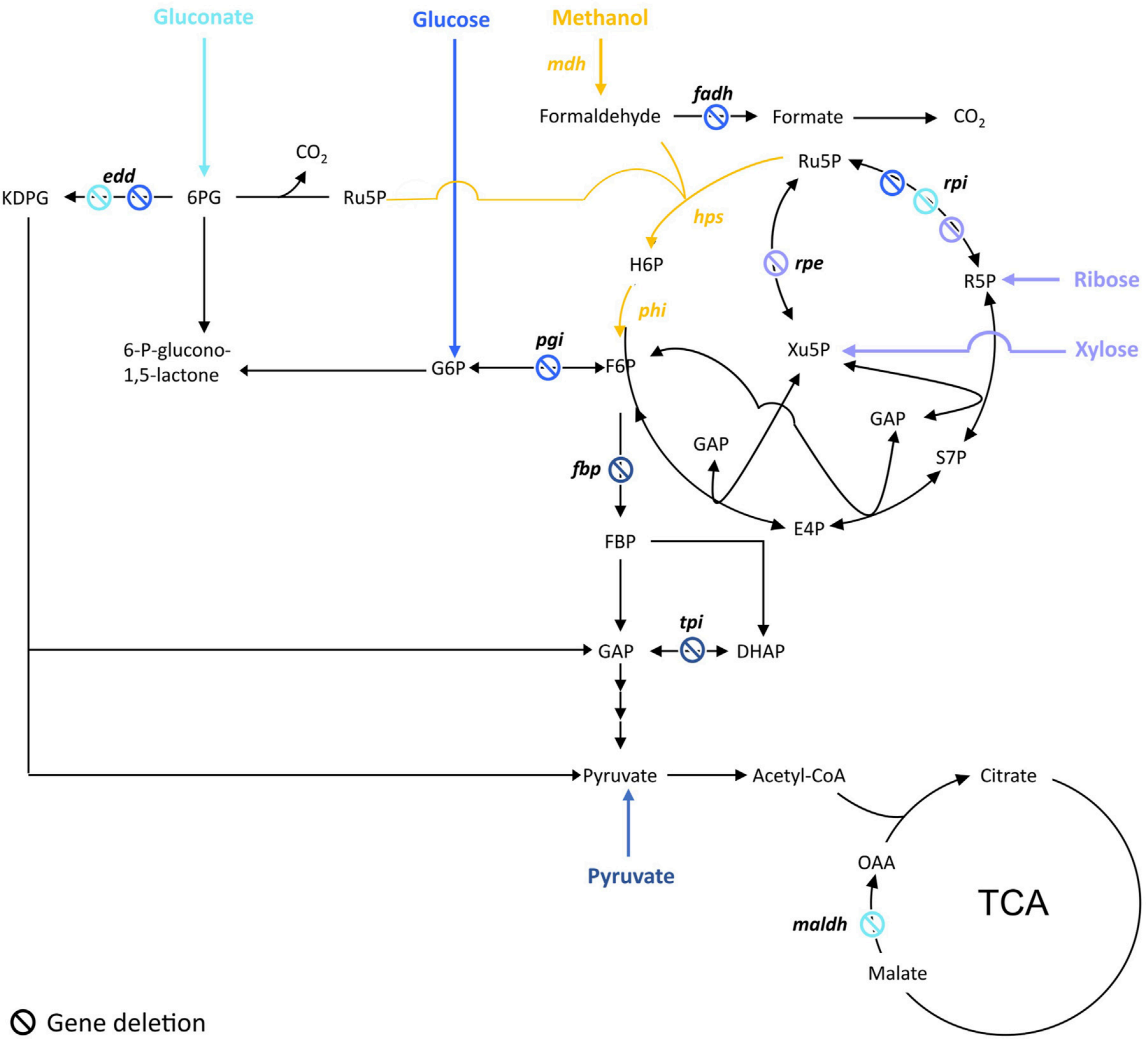
Targeted genes to construct methanol-dependent strains in *E. coli*. Adapted from (Wang et al., 2020b). Engineered synthetic methanol-dependent strain co-assimilating gluconate (in light blue) by (Meyer, et al., 2018); co-assimilating glucose (in blue) by (Bennett, et al., 2020b); co-assimilating ribose by (Chen, et al., 2018) or xylose by (Chen, et al., 2018; Chen, et al., 2020) (in purple); co-assimilating pyruvate (in dark blue) by (Keller, et al., 2020). Enzymes are written in bold. edd, 6-phosphogluconate isomerase; fadh, formaldehyde dehydrogenase; hps, 3-hexulose-6-phosphate synthase; maldh, malate dehydrogenase; mdh, methanol dehydrogenase; pgi, glucose-6-phosphate isomerase; phi, 6-phospho-3-hexuloisomerase; rpe, ribulose-3-phosphate epimerase; rpi, ribose-5-phosphate isomerase. Ac-CoA, acetyl-CoA; E4P, erythrose-4-phosphate; F6P, fructose-6-phosphate; FBP, fructose-1,6-biphosphate; GAP, glyceraldehyde-3-phosphate; DHAP, dihydroxyacetone-phosphate, H6P, hexulose-6-phosphate; KDPG, 2-keto-3-deoxy-6-phosphogluconate; 6 PG, 6-phosphogluconate; R5P, ribose-5-phosphate; Ru5P, ribulose-5-phosphate; S7P, sedoheptulose-7-phosphate; SBP, sedoheptulose-1,7-biphosphate; Xu5P, xylulose-5-phosphate.

To ensure methanol is co-utilized with the other carbon source, cells are engineered in order to gain a benefit in utilizing methanol. To do so, the common strategy was to design a strain dependent on methanol utilization for growth while the other carbon source is used only to ensure Ru5P production. This is achieved by blocking either the conversion of glucose to F6P or the Ru5P catabolic pathways depending on the carbon source used (Figure 1). That way, cells have no other choice than producing F6P from the co-assimilation of methanol and the other carbon source while ensuring a high pool of the key Ru5P. This strategy was applied by Meyer et al. to engineer an *E. coli* methanol dependent-strain (∆*rpiAB*∆*edd*∆*maldh*) co-utilizing gluconate (Meyer, et al., 2018). Similarly, Chen et al. engineered an *E. coli* strain (∆*rpiAB*) co-utilizing xylose and another one (∆*rpe*) co-utilizing ribose (Chen, et al., 2018). Later, to build their fully methylotrophic strain, Chen et al. adopted the same strategy than in 2018 by deleting *rpiAB* but switched from *E*. *coli* BL21 to *E*. *coli* K12 BW25113 to facilitate the genetic engineering efforts (Chen, et al., 2020). To enable the co-utilization of methanol and glucose, Bennet et al. first deleted the phosphoglucose isomerase (*pgi*) that converts glucose-6-phosphate (G6P) to F6P, so that G6P is only used by the oxidative part of the PPP to directly fill the pool of Ru5P. The *frmA* gene, encoding for formaldehyde dehydrogenase, was also deleted in order to push formaldehyde towards assimilation (Bennett, et al., 2018). Later, authors went ahead by knocking-out *edd* and *rpiAB* in their ∆*frmA*∆*pgi E. coli* strain to completely abolish glucose assimilation but amino acids supplementation in the cultivation medium was then required (Bennett, et al., 2020b). Keller et al. used a different strategy by applying first a flux balance analysis and identifying candidate deletions *in silico* that could lead to methanol-auxotrophy with a complete RuMP cycle and a high degree of methanol incorporation. Authors investigated two out of 1,200 candidate strains, one with a deletion of fructose-1,6-bisphosphatase (*fbp*) and another with triosephosphate isomerase (*tpiA*) deleted. Those strains were methanol dependent and showed a 99% fractional incorporation of methanol into RuMP cycle metabolites (Keller, et al., 2020).

If most of the attempts were done in *E. coli*, methanol dependent strains were also obtained in *C. glutamicum* by generating ∆*aldh*∆*fadH*∆*rpi* and ∆*aldh*∆*fadH*∆*rpe* mutants*,* respectively using xylose or gluconate and ribose or gluconate as co-substrates (Hennig et al., 2020; Tuyishime et al., 2020). The *aldH* and *fadH* genes are respectively coding for an acetaldehyde dehydrogenase and a formaldehyde dehydrogenase, so their deletion prevented formaldehyde oxidation to CO_2_. More recently, synthetic methanol auxotrophy has also been engineered in *Bacillus subtilis* by deleting *rpiAB* and *rpe* (Gao et al., 2022).

##### Adaptive Laboratory Evolution

By engineering methanol-dependent strains, methanol assimilation is coupled with cell growth thus opening the way to ALE to improve methanol assimilation. ALE is used on microorganisms cultivated under defined conditions for weeks to months in order to select improved phenotypes on relevant carbon sources. Microorganisms can either be cultured in shake flasks with sequential serial passages or cells can be grown in chemostats with a controlled environment in which one component of the medium is limiting. Shake flasks have the great advantage to enable to run many culture conditions in parallel but pH, oxygenation and cell density can vary during the experiment. By using a chemostat, these parameters as well as growth rate can be closely monitored and kept constant, and higher cell densities can be reached. However, the operation costs and the know-how of this device are higher than for shake flasks (Dragosits et al., 2013).

Mutants evolving towards a faster growth can be selected, and methanol utilization can be tested by measuring the incorporation of label in intracellular metabolites from ^13^C-methanol (Table 1). Meyer et al. decided to knock out *maldh* in a ∆*rpiAB*∆*edd* background after *in silico* analysis, and to apply ALE to this *E*. *coli* strain. A mutant co-utilizing methanol and gluconate at a growth rate of µ = 0.08 h^−1^ and with a methanol uptake rate of 13 mmol. gCDW^−1^. h^−1^ - which is close to the one reported for natural methylotrophs—was selected. In this condition, 21% of the PEP carbon atoms came from methanol (Meyer, et al., 2018). When Chen et al. used ALE on their ∆*rpiAB* strain, they succeeded to select a strain with a growth rate of µ = 0.17 h^−1^. This strain was able to produce butanol from the co-consumption of methanol and xylose, and 22% of butanol carbons came from methanol (Chen, et al., 2018). After the ALE experiment, Bennet et al. isolated an *E. coli* strain that was growing on glucose and methanol with a specific growth rate of µ = 0.15 h^−1^ and without the need for amino acid supplementation. The strain was able to produce acetone, for which 22% of carbon atoms derived from methanol (Bennett, et al., 2020b). An ALE experiment was successful for Chen et al. as they obtained an evolved methylotrophic *E. coli* strain that exhibited a growth rate of µ = 0.08 h^−1^ on pure methanol (Chen, et al., 2020). However, no proof of *in vivo* functionality of the introduced methanol pathway was shown.

**TABLE 1:** Overview of ^13^C-methanol incorporation level at the intracellular level or in final products in strains after ALE experiment.

Organism	Cultivations Conditions	^13^C-Enrichment	References
*E. coli* BW25113	500 mM ^13^C-methanol + 5 mM gluconate	21% in PEP	Meyer, et al. (2018)
*E. coli* BL21 DE3	250 mM ^13^C-methanol + 50 mM xylose	22% in butanol	Chen, et al. (2018)
*E. coli* BW25113	500 mM ^13^C-methanol + 200 mM glucose	22% in acetone	Bennett, et al. (2020b)
*C. glutamicum* ATCC 13032	125 mM ^13^C-methanol + 27 mM xylose	22% in PEP	Tuyishime, et al. (2018)
*C. glutamicum* ATCC 13032	469 mM ^13^C-methanol + 27 mM xylose	20–30% in amino acids	Wang, et al. (2020a)
*C. glutamicum* ATCC 13032	500 mM ^13^C-methanol +20 mM gluconate+ 0.5 g/L yeast extract	30% in cadaverine	Hennig, et al. (2020)

Tuyshime et al. performed ALE experiments on their *C. glutamicum* mutant and selected a strain growing on xylose and methanol at a growth rate of 0.03 h^−1^ with a methanol uptake rate of 0.86 mmol. gCDW^−1^. h^−1^ (Tuyishime et al., 2018). Based on transcriptome analysis, further work was done on this strain to explore the metabolic regulation that operated during methylotrophic conditions (Fan et al., 2021). Results demonstrated that the evolved *C. glutamicum* used the SBPase variant to recycle Ru5P as *fba* and *glpX* were upregulated while *tal* was downregulated. Nitrate was also shown to serve as a complementary electron acceptor during aerobic methanol metabolism as the operon *narKGHJI* was upregulated. This operon encodes for a respiratory nitrate reductase which is usually active during anaerobic growth. Amino acids biosynthesis was also shown to limit growth on methanol as genes involved in the biosynthesis of several L-aspartate derived amino acids were downregulated. This strain was subjected to a second ALE experiment which enabled to select a new evolved strain exhibiting a growth rate of 0.052 h^−1^ on xylose and methanol as well as a higher tolerance towards methanol (Wang et al., 2020a). In another study, Henning et al. selected, in *C. glutamicum*, an evolved ∆*aldh*∆*fadH*∆*rpe* strain co-utilizing methanol and gluconate. This strain was able to produce cadaverine, for which 43% of carbon atoms came from methanol. After a new round of ALE experiments started from the previous strain, another one was selected on ribose and methanol. This strain grew without yeast extract and exhibited a specific growth rate of µ = 0.10 h^−1^ (Hennig, et al., 2020).

##### Resulting Metabolic Adaptation After ALE

As in nature, during ALE genomic changes occur to generate an improved phenotype enabling cells to cope with their environment. It is of great interest to get insight on which genes are essential (or non-essential) for methanol assimilation by investigating the mutations and their consequences. Mutations found in evolved strains can be classified in three categories, mutations affecting: 1) the dissimilatory pathway and the recycling pathways; 2) the redox and energy balance; 3) the substrate uptake and other enzymes connected to metabolism (Wang et al., 2020b).

Mutations in the *frm* operon genes leading to the inactivation of the formaldehyde dissimilatory pathway were found in all the evolved *E. coli* strains (Chen, et al., 2018; Meyer, et al., 2018; Chen, et al., 2020). These mutations are consistent with the fact that efficient methanol-dependent growth required formaldehyde to be entirely redirected towards C1-assimilation. Mutations leading to *pykF* and *zwf* inactivation possibly enabled to increase the F6P pool size for Ru5P regeneration via the PPP (Chen et al., 2018). Similarly, the *deoD* mutation was thought to increase the Ru5P pool size for formaldehyde assimilation (Chen, et al., 2018).

Mutations were found in *nadR* (Meyer, et al., 2018), *cyaA* (Chen, et al., 2018) in *E. coli* and *mtrA* in *C. glutamicum* (Tuyishime, et al., 2018). The enzymes encoded by these genes are involved in the redox state or in the energy supply of the cell. The *nadR* and *mtrA* genes encode for enzymes involved in the balance of the redox state. Both mutations are thought to lead to the increase of NAD^+^ availability in the cell, thereby most probably promoting methanol oxidation. *cyaA* encodes for an enzyme producing cyclic AMP (cAMP) from ATP. The transcription of most of the TCA cycle enzymes is activated by cAMP. Most probably a *cyaA* mutation would lower the TCA cycle activity and consequently NAD(P)H production, at a beneficial cost for methanol oxidation. Moreover, inactivation of *frmA* and *fdoG* (encoding a formate dehydrogenase) for the synthetic methylotrophic strain, help limiting further the production of NADH (Chen, et al., 2020).

Mutations in *gnd* encoding for 6-phosphogluconate dehydrogenase were found in a strain assimilating methanol better than the parental one but still requiring xylose for growth (Chen, et al., 2020). GntR and AltR are transcriptional regulators and mutations in genes encoding these proteins were found respectively in the *E. coli* strain co-utilizing methanol and gluconate and *C. glutamicum* strain co-utilizing methanol and xylose (Meyer, et al., 2018; Tuyishime, et al., 2018). GntR represses two enzymes involved in gluconate uptake, therefore *gntR* mutations may have improved gluconate uptake. Similarly, AltR regulates among other genes the expression of *xylB* encoding for a xylulose kinase and *adhA* encoding for an alcohol dehydrogenase functioning as a methanol dehydrogenase in *C. glutamicum*. Therefore, an *altR* mutation may have modified *xylB* and *adhA* expression leading to improved co-utilization of methanol and xylose by increasing substrate uptake. In the strain exhibiting higher tolerance towards methanol, two mutations in *cgl0653* and *cgl0833,* respectively encoding for an *O-*acetyl-L-homoserine sulfhydrylase and for a methanol-induced membrane-bound transporter were found (Wang, et al., 2020a).

#### Spatial Engineering

Once produced, formaldehyde has to be quickly condensed with Ru5P to avoid its toxic effect. To optimize formaldehyde assimilation, another strategy has been proposed that consists in enhancing substrate channelling by co-localizing the enzymes in a delimited space. Orita et al. were the first to address this question by fusing *hps* and *phi* from *Mycobacterium gastri* (Orita et al., 2007). *In vivo* assays showed that when expressed in *E. coli*, the fusion protein of Hps and Phi leads to an increased growth rate on formaldehyde than when the proteins were separated. Later, Price et al. advanced the field by engineering a supramolecular enzyme complex with Mdh from *B. methanolicus* and Hps and Phi from *Mycobacterium gastri* (Price, et al., 2016). They took advantage of the decameric structure of Mdh to design a supramolecular complex able to self-assemble by using SH3-ligand in order to “plug” on it the fusion protein of Hps and Phi previously described by Orita et al. This engineered complex enabled a faster conversion of methanol into F6P compared to unassembled proteins *in vitro*. In the same way, Fan et al. used an alternative strategy to fuse Mdh from *B. stearothermophilus* with Hps and Phi from *B. methanolicus* by using flexible linkers (GGGGS)_n_. They also demonstrated an improvement in methanol conversion to F6P *in vitro* (Fan, et al., 2018).

### The XuMP Pathway

Dai et al. introduced the XuMP pathway in the non-methylotrophic yeast *S. cerevisae* by integrating an alcohol oxidase, a catalase, a dihydroxyacetone synthase (Das) and a dihydroxyacetone kinase (DhaK) from *Pichia pastoris* in its genome (Dai, et al., 2017). By using the native peroxisome targeting peptide of *P. pastoris*, enzymes were addressed to the *S. cerevisae* peroxisome. When yeast extract was added to the medium, methanol consumption was further improved from 1.04 g/L to 2.35 g/L, suggesting that Xu5P recycling was enhanced by yeast extract addition. However, no proof of *in vivo* functionality of the introduced methanol pathway was shown.

In the yeast *Y. lipolitica*, the expression of a dihydroxyacetone synthase from *Candida boidini* restored the formaldehyde tolerance of a formaldehyde sensitive strain (i.e. deleted for the FaDH) but was not enough to convert methanol into biomass (Vartiainen, et al., 2019).

## Mix and Match Enzymes

**FIGURE 2:**
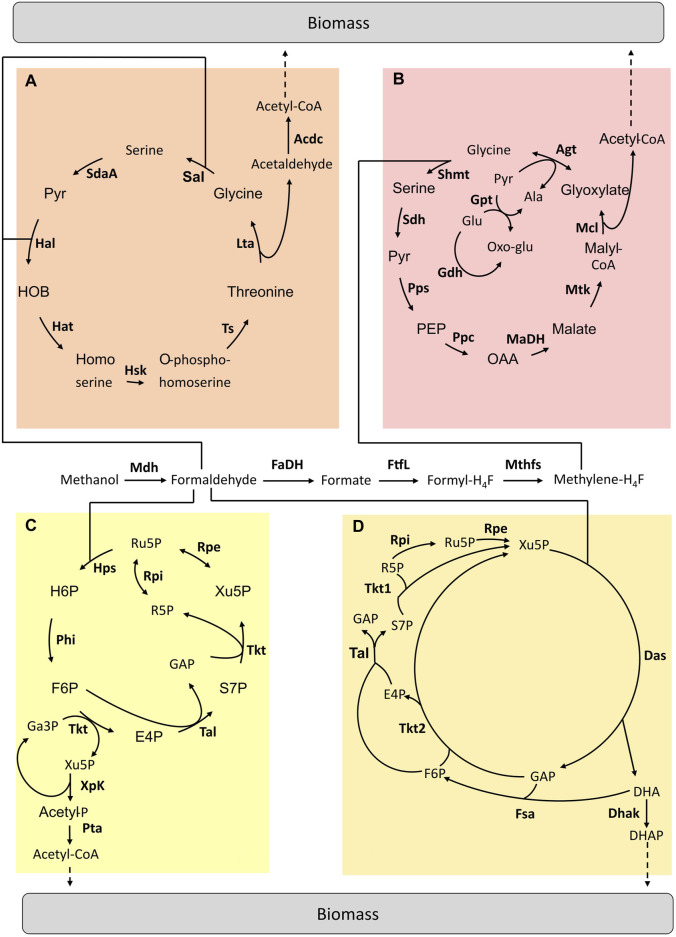
Hybrid pathways implemented for C1-assimilation. **(A)** Homoserine cycle pathway **(B)** Modified serine cycle pathway **(C)** Methanol condensation cycle pathway **(D)** Hybrid Mdh-Das pathway. Adapted from (Tuyishime, et al., 2020). Enzymes are written in bold. Acdc, acetaldehyde dehydrogenase; Agt, Alanine-glyoxylate aminotransferase; Dhak, DHA kinase; FaDH, formaldehyde dehydrogenase; FtfL, formate-H_4_F ligase; Fsa, Fructose-6-aldolase; Gdh, glutamate dehydrogenase; Gpt; Glutamate-pyruvate transaminase; Hal, 4-hydroxy-2-oxobutanoate aldolase; Hat, 4-hydroxy-2-oxobutanoate aminotransferase; Hsk, homoserine kinase; Lta, threonine aldolase; MaDH, malate dehydrogenase; Mcl, malyl-CoA lyase; Mdh, methanol dehydrogenase; Mthfs, methylene-H_4_F dehydrogenase; Mtk, malate thiokinase; Ppc, phosphoenolpyruvate carboxylase; Pps, phosphoenolpyruvate synthetase; Rpe, ribulose-3-phosphate epimerase; Rpi, ribose-5-phosphate isomerase; Sal, serine aldolase; SdaA, serine deaminase; Sdh, serine, dehydratase; Shmt, serine-H_4_F hydroxymethyltransferase; Tal, transaldolase; Tkt1 & Tkt2, transketolase type 1 & 2; Ts, threonine synthase; Ala, alanine; E4P, erythrose-4-phosphate; F6P, fructose-6-phosphate; GAP, glyceraldehyde-3-phosphate; Glu, glutamate; H6P, hexulose-6-phosphate; OAA, oxaloacetate; Oxo-Glu, 2-oxoglutarate; PEP, phosphoenolpyruvate; Pyr, pyruvate; R5P, ribose-5-phosphate; Ru5P, ribulose-5-phosphate; S7P, sedoheptulose-7-phosphate; SBP, sedoheptulose-1,7-biphosphate; Xu5P, xylulose-5-phosphate.

Different from the above strategies that mainly build on existing pathways, the “mix and match” approach allowed combining existing enzymes from different microbial sources into a synthetic pathway and implementing it in a host. Considering the methylotrophic metabolic diversity, we can estimate that there are more than 500 unique methanol assimilation pathways from methanol to biomass (Heux, et al., 2018). Therefore, such an approach is perfectly adapted to explore this large space of metabolic solutions while identifying the most powerful combinations. At the moment, only four hybrid pathways have been studied (Figure 2).

### The Serine and Homoserine Cycles

Yu and Liao implemented a modified serine cycle in *E. coli* (Figure 2B)*.* Methanol is assimilated via the H_4_F-dependent pathway by being converted into formate. Rather than “copy-pasting” the serine cycle, the authors decided to select a set of enzymes to replace key reactions in the natural pathway by other known reactions from other microorganisms to fit the activity of this pathway with *E. coli* metabolism. The *E. coli* metabolic network was modified to become dependent on methanol for growth. Once the operation of the two modules was confirmed (i.e. formate conversion to CH_2_H_4_F and the modified serine cycle), the complete pathway was tested *in vivo* by using ^13^C-methanol and xylose. Label derived from methanol was found in intracellular metabolites (Yu et al., 2018). Later, He et al. designed a complete homoserine cycle based on *E. coli* native enzymes and the promiscuous activity of the formaldehyde aldolase (Figure 2A). Unlike the serine cycle, here the C1 intermediate for assimilation is formaldehyde. A codon-optimized Mdh from *C. glutamicum* was used to oxidise methanol to formaldehyde. Then, the *E. coli* native metabolic network was modified in order to design a strain in which methanol assimilation is required for serine biosynthesis. The functionality of the homoserine cycle was tested *in vivo* with labelling experiments by using *E. coli* auxotrophic strains in which the complete formaldehyde dissimilation pathway was blocked. However, optimization is still required to achieve *E. coli* growth using the homoserine cycle (He et al., 2020).

### The Methanol Condensation Cycle

In 2014, Bogorad et al. designed a non-natural pathway enabling methanol assimilation named methanol condensation cycle (MCC) (Figure 2C) (Bogorad et al., 2014). In the MCC pathway, existing enzymes from various microorganisms were combined to convert methanol into acetyl-CoA. These enzymes do not work together in nature. The MCC pathway was first constructed *in silico* and then tested in a cell-free environment. This pathway ensures the conservation of phosphates in the catalytic cycle as well as the balance of the redox state. The MCC was found to be functional and acetyl-CoA production was achieved *in vitro*. Acetyl-CoA is a precursor for the production of biofuels (i.e. ethanol and butanol). However, there is no report of the *in vivo* functionality of the MCC pathway yet.

### The Hybrid Mdh-Das Pathway

The hybrid Mdh-Das (HMD) pathway (Figure 2D) was built in *E. coli* using an iterative combination of dry and wet lab approaches to design, implement and optimize this metabolic trait (De Simone, et al., 2020). Through *in silico* modelling, a new route that “mixed and matched” two methylotrophic enzymes, a bacterial methanol dehydrogenase (Mdh) and a dihydroxyacetone synthase (Das) from yeast was designed. To identify the best combination of enzymes to introduce into *E. coli*, a library of 266 pathway variants containing different combinations of Mdh and Das homologues was built and then screened using high-throughput ^13^C-labeling experiments. The highest level of incorporation of methanol into central metabolism intermediates (i.e. 22% into PEP) was obtained using a variant composed of a Mdh from *Acinetobacter gerneri* and a codon-optimized version of *Pichia angusta* Das. Finally, the activity of the HMD pathway was further improved by engineering strategic metabolic targets identified using omics and modelling approaches. The final synthetic strain had 1.5 to 5.9 times higher methanol assimilation in intracellular metabolites and proteinogenic amino acids than the starting strain but was still unable to grow on methanol as the sole carbon and energy source (De Simone, et al., 2020).

A chimeric methanol assimilation pathway was engineered in *Y. lipolytica* by introducing both the RuMP and the XumP pathways (Wang et al., 2021). In this strain, methanol was oxidised by the Mdh from *B. stearothermophilus*. For the RuMP pathway, Hps and Phi from *B. methanolicus* were selected while Das and DhaK from *P. pastoris* were chosen. The formaldehyde dissimilation pathway was blocked by deleting *FLD1* the gene, the homologue of *frmA* in *E. coli*. Recycling of the C1-acceptors, Ru5P and Xu5P, was enhanced by the overexpression of several key genes of the glycolysis and the PPP (i.e. *TKL1*, *PFK*, *FBA*, and *RPE1*) as well as *glpX* from *B. methanolicus* that encodes for a SBPase and a FBPase. After ALE experiments, two evolved strains with improved methanol assimilation were obtained. Interestingly, in one strain, only the expression of the genes of the RuMP pathway was upregulated while in the other one the expression of both RuMP and XuMP pathways was increased. This highlights the capacity of the microbial strain to invoke several strategies in response to externally introduced genetic perturbations.

## Create Novel Enzymes

**FIGURE 3:**
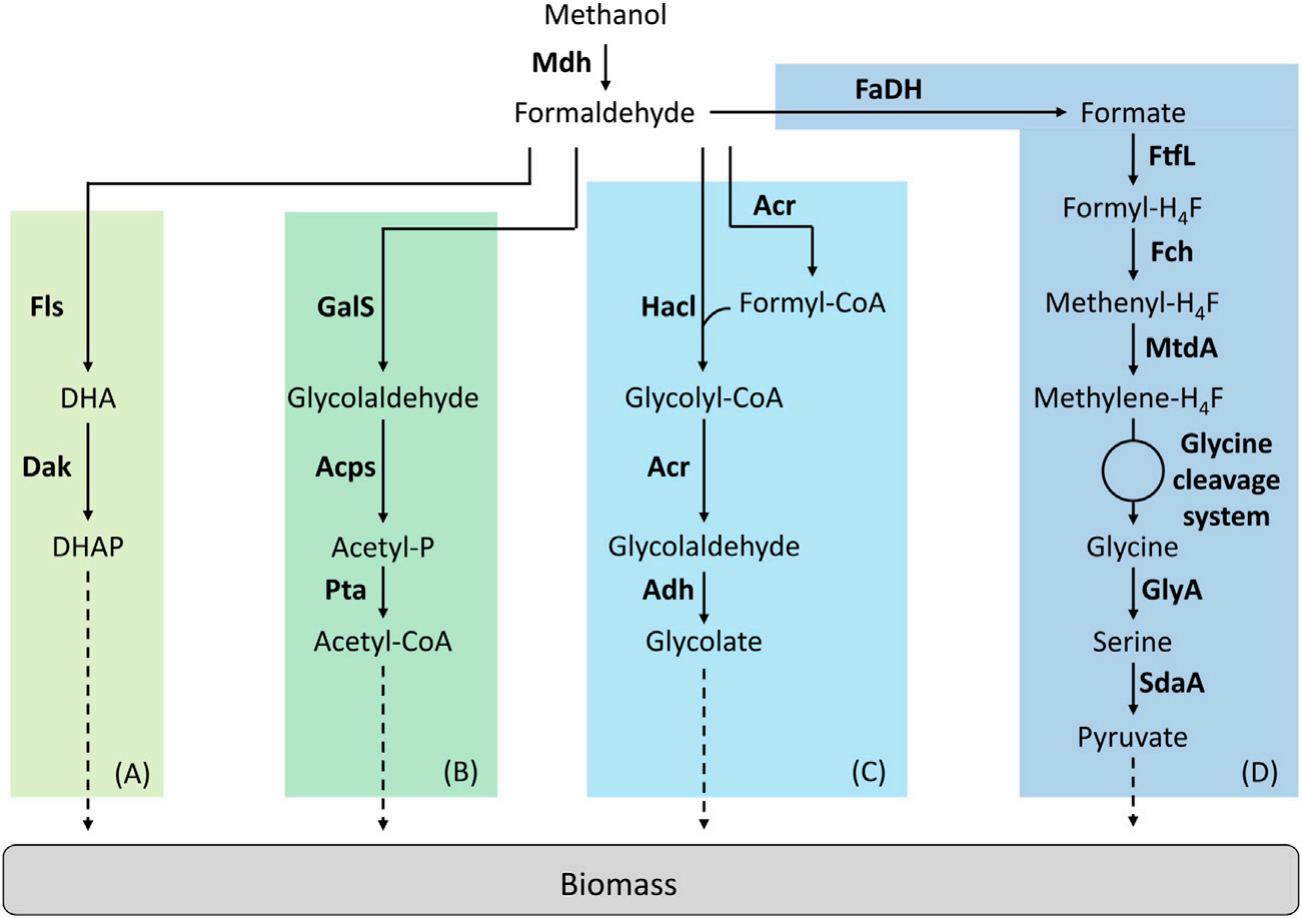
Synthetic linear pathways implemented for C1-assimilation. **(A)** Formolase pathway, **(B)** Synthetic acetyl-CoA pathway, **(C)** 2-hydroxyacyl-CoA lyase pathway, **(D)** Reductive glycine pathway. Adapted from (Tuyishime, et al., 2020). Enzymes are written in bold. Acps, acetylphosphate synthase; Acr, acyl-CoA reductase; Adh, aldehyde dehydrogenase; Dak, dihydroxyacetone kinase; FaDH, formaldehyde dehydrogenase; Fch, methenyl H_4_F-cyclohydrolase; Fls, formolase; FtfL, formate-H_4_F ligase; GalS, glycolaldehyde synthase; GlyA, serine hydroxymethyltransferase; Hacl, 2-hydroxyacyl-CoA lyase; Mdh, methanol dehydrogenase; MtdA, methylene-H_4_F dehydrogenase/methylene-H_4_MPT dehydrogenase; Pta, phosphate acetyltransferase; SdaA, serine deaminase. DHA, dihydroxyacetone; DHAP, dihydroxyacetone phosphate.

Creating new reactions that do not exist in nature is the ultimate strategy to fully explore the metabolic solution space. With the advances in enzyme engineering and *de novo*-enzyme design, it becomes possible to create these reactions and integrate them into a synthetic pathway to perform a novel function. So far, only four studies have implemented synthetic methylotrophic pathways that involve novel catalytic transformations (Figure 3).

### The Formolase Pathway

Wang et al. were the first in succeeding to implement a linear synthetic pathway. This pathway relies on two enzymes, a NAD-dependent Mdh and a formolase (Fls) (Figure 3A). Fls is a synthetic, computationally designed enzyme (Siegel, et al., 2015) that condensates three molecules of formaldehyde to produce one molecule of DHA. The cooperation of the Mdh from *B. methanolicus* MGA3 or PB1 were first tested with Fls *in vitro*. From this experiment, authors decided to introduce the Mdh from *B. methanolicus* PB1 combined with Fls in an *E. coli* strain deleted for *frmA*. To improve the performance of this strain, ALE was used. By using ^13^C-methanol, the resulting evolved strain was shown to have a higher level of labelling in the proteinogenic amino acids than the parental strain but failed to growth on pure methanol (Wang et al., 2017).

### The Synthetic Acetyl-CoA and Glycolaldehyde Assimilation Pathway

The synthetic acetyl-CoA (SACA) is a linear pathway that was designed and constructed by Lu et al. (Figure 3B). This pathway was first designed to produce acetyl-CoA from formaldehyde and relies on three enzymes. A glycolaldehyde synthase (GalS) condensates two molecules of formaldehyde to produce one molecule of glycolaldehyde. Then, glycolaldehyde is converted into acetyl-phosphate by the acetyl-phosphate synthase. Acetyl-phosphate is subsequently used to produce acetyl-CoA by the phosphate acetyltransferase. The authors first designed GalS and checked for glycolaldehyde production. Then, they optimized GalS by using directed evolution to improve the kinetic properties of the enzyme. The SACA pathway was functional *in vitro* and the authors then decided to implement the pathway in *E. coli*. The functionality of the pathway was tested *in vivo* in rich media. By combining the SACA pathway with the Mdh from *B. stearothermophilus*, a slight improvement in the final OD was observed. When tested on minimal media by using ^13^C-methanol, label was also found in some proteinogenic amino acids after 26 h but no growth was observed (Lu et al., 2019).

Based on a computational analysis of metabolic reactions from MetaCyc and Atlas databases, Yang et al. designed a new formaldehyde assimilation pathway named the glycolaldehyde assimilation (GAA) pathway (Yang et al., 2019). This pathway was tested *in vitro* using engineered versions of GalS and transaldolase B and reached a carbon yield of 88%. However, no proof of *in vivo* functionality of this pathway was shown. More recently a new GalS was engineered based on a newly discovered glyoxylate carboligase enzyme found in *E. coli* (Jo et al., 2022). One of the variants showed a 10 times higher affinity and a 2 times higher catalytic efficiency for formaldehyde compared to the previously described GalS (Lu, et al., 2019; Yang, et al., 2019). When this optimized GalS was associated *in vitro* to a lactaldehyde reductase (FucO)*,* 66% of the formaldehyde was converted into ethylene glycol (Jo, et al., 2022).

### The 2-Hydroxyacyl-CoA Lyase Pathway

The 2-hydroxyacyl-CoA lyase (Hacl) is a synthetic pathway designed by Chou et al. where Hacl condenses formaldehyde with formyl-CoA to produce glycolyl-CoA (Figure 3C). Glycolyl-CoA is subsequently converted to glycolaldehyde by an acyl-CoA reductase (Acr). Finally, a aldehyde dehydrogenase produces glycolate from glycolaldehyde. Hacl is a mammalian enzyme involved in α-oxidation. However, after a BLAST research limited to prokaryotes, the authors identified and tested one Hacl from *Rhodospiralleles bacterium* (RuHacl) exhibiting the condensation activity of formaldehyde with formyl-CoA. They tested the pathway *in vivo* in an *E. coli* strain deleted for ∆*frmA,* ∆*fdhF*∆*fdnG*∆*fdoG* and ∆*glcD* to avoid any competitive reactions using formaldehyde, formate or gluconate. The strain produced 0.5 g/L of glycolate corresponding to a yield of 67%. After enzyme engineering using directed evolution, a variant of RuHacl with improved enzyme kinetics was selected. By expressing this variant, *E. coli* produced 1.2 g/L of glycolate. However, the pathway has not been tested yet *in vitro* or *in vivo* on methanol with the addition of a Mdh (Chou et al., 2019). Later, the Hacl pathway was used to establish the formyl-CoA elongation (FORCE) pathway (Chou et al., 2021). The FORCE pathway was designed as an orthogonal platform for C1 utilization. Based on thermodynamics and stoichiometric analyses, different FORCE pathways were evaluated. Several were tested both *in vitro* and *in vivo* in *E. coli*. The conversion of formate, formaldehyde and methanol into glycolate, ethylene glycol and glycerate was demonstrated among other products.

It was shown that Hacls were poorly produced in *E. coli*, which could limit its efficiency (Chou, et al., 2019; Burgener et al., 2020). Together with Hacl, Oxalyl-CoA decarboxylase (Oxc) are members of a family enzyme catalyzing the condensation of formyl-CoA with formaldehyde to produce glycolyl-CoA. Oxc was thus repurposed to improve its glycolyl-CoA synthase activity under physiological conditions (Nattermann et al., 2021). Oxc from *Methylorubrum extorquens* (MeOxc) was subjected to several rounds of iterative site mutagenesis. A quadruple variant MeOxc4 was selected showing affinity towards formaldehyde similar to natural formaldehyde converting-enzymes and with an improvement for the carboligation activity of 200-fold compared to wild-type MeOxc. To test this enzyme *in vivo*, RuHacl was replaced with MeOxc4 in the *E. coli* strain previously designed for whole-cell bioconversion (Chou, et al., 2019; Nattermann, et al., 2021). Glycolate production was two-fold higher using MeOxc4 than RuHacl. With higher production rates in *E. coli*, this enzyme offers great possibilities for the engineering of new linear C1 pathways.

### The Reductive Glycine Pathway

Kim et al. designed a fourth linear pathway: the reductive glycine pathway which, combined with Mdh, enabled methanol assimilation in *E. coli* (Figure 3D). In this pathway, formate is the key C1-intermediate that enables carbon assimilation (Kim, et al., 2020). The authors introduced the H_4_F-pathway from *Methylobacter extorquens* associated with a glycine cleavage system in an *E. coli* strain auxotrophic for serine, glycine and C1 moieties (∆*serA*∆*kbl*∆*ltaE*∆*aceA*). It has been previously described that even if growth could not be supported on formate, the latter compound could be still assimilated in *E. coli* via the H_4_F-dependent pathway (Tashiro et al., 2018). Kim et al. optimized the operation of this pathway first on formate. Then, the authors optimized the strain using ALE before implementing Mdh. The selected strain was able to grow on formate and CO_2_ at a growth rate of µ = 0.086 h^−1^. In this strain, methanol to be assimilated is first oxidized to formaldehyde by Mdh and then formaldehyde is oxidized to formate *via* the native GSH-dependent pathway of *E. coli* encoded by the operon *frmRAB*. Several Mdhs were tested and only the Mdh from *B. stearothermophilus* supported growth on methanol and CO_2_ at a growth rate of µ = 0.013 h^−1^. To confirm that the methanol pathway was functional, they confirmed that a *frmA* deletion blocked growth on methanol. By using ^13^C-methanol, authors showed that methanol carbon atoms were recovered in proteinogenic amino acids. The labelling pattern found was the same than when the strain grew on ^13^C-formate and ^12^CO_2_.

## Summary and Perspectives

Among all the attempts to engineer synthetic methylotrophy in the platform host microorganisms cited above, it is very interesting to notice that synthetic methylotrophic growth was only achieved in *E. coli* and using both a natural cyclic pathway (Chen, et al., 2020) and a synthetic linear methylotrophic pathway (Kim, et al., 2020). In both examples, rational and evolutionary approaches have been combined. Even if different NAD-dependent Mdhs were used, both are exhibiting improved kinetic parameters for methanol oxidation (i.e. engineered version of Mdh from *C. necator* (Chen, et al., 2020) and Mdh from *B. stearothermophilus* (Kim, et al., 2020)). Moreover, in both cases, auxotrophic strains were first designed prior to applying ALE, which has been determinant to reach the final phenotype. Indeed, when comparing the flux distribution within the metabolic network of the native methylotroph *B. methanolicus* in both methylotrophic and non-methylotrophic conditions (Delepine et al., 2020), we quickly realized that the number of metabolic adaptations required to enable growth on methanol is too high to be solely achieved by a targeted engineering approach. ALE thus appears as the most reasonable strategy to achieve the complete reorganisation of the central metabolism required to fit a methylotrophic lifestyle. In all these studies (Chen, et al., 2018; Meyer, et al., 2018; Bennett, et al., 2020b; Chen, et al., 2020; Keller, et al., 2020), ALE helped in rerouting carbon fluxes towards methanol assimilation by limiting competing pathways and improving substrate uptakes in order to enhance efficient biomass production while adjusting the energy and redox state of the cell. Nevertheless, sometimes rational engineering was necessary in between two rounds of ALE to obtain a fully methylotrophic lifestyle (Chen, et al., 2020). The length of ALE varied according to the study but at least 6 months were necessary to select a strain growing solely on methanol. When looking at the genetic adaptations level, ALE revealed that some are very specific of the co-assimilated carbon source used while others are more general, e.g. the mutations in the genes involved in redox homeostasis or formaldehyde detoxification. It would be very interesting to reconstruct a strain with those mutations in order to identify the minimal set of mutations required for building a true synthetic methylotroph.

However, despite these encouraging accomplishments, synthetic methylotrophy remains a major challenge as the observed growth rates are largely sub-optimal and can barely be considered as active growth. Doubling times of 50 h on a mixture of methanol-CO_2_ and 9 h on methanol (corresponding to growth rates of 0.01 and 0.08 h^−1^, respectively) are far from the performance of natural methylotrophs that is 0.20 h^−1^ for *Bacillus methanollicus* at 37°C (Müller et al., 2014); 0.10–0.15 h^−1^ for *Pichia Pastoris* at 25°C (Tomàs-Gamisans et al., 2018); and 0.17 h^−1^ for *M. extorquens* at 28°C (Kiefer et al., 2009). Both strains are not yet fully adapted to these non-native carbon sources and further work is necessary. So far, efforts to engineer this non-native substrate catabolism have all taken the straightforward approach of metabolic pathway overexpression, ignoring coordination with the overall cellular processes that include central metabolism, stress-response and cell doubling. However, natural systems use genome-scale regulatory networks, called regulons, which coordinate nutrient catabolism with the larger cellular infrastructure. Therefore, nutrient metabolism cannot be considered as a hermetic process and engineering non-native carbon source utilisation should promote regulation processes together with metabolic pathway diversification and improvement for an efficient and dynamic cellular coordination. Indeed, recent studies demonstrated that targeting regulation of amino acid biosynthesis, an essential process to biomass formation and which is negatively impacted when *E. coli* grows on methanol, can improve methanol assimilation levels (Bennett et al., 2020a; Bennett et al., 2021).

In addition, synthetic biologists have left out the exploration of the spatial dimension for metabolic engineering in prokaryotes, as they do not have any particular subcellular organization. However, a study revealed that around 17% of bacteria contain a bacterial microcompartment (BMC) locus in their genome and that microcompartments are found in 23 different phyla (Axen et al., 2014) including *E. coli*. In addition, compartmentalization of the methanol assimilation pathway in peroxisomes has proven to be efficient in methylotrophic yeasts to protect themselves against toxicity of reactive intermediates (i.e. formaldehyde and H_2_O_2_) while improving the reaction efficiencies as enzymes are in close vicinity to their substrates and intermediates (van der Klei et al., 2006). Furthermore, compartmentalization allows to build an orthogonal network structure operating with minimal interactions to native metabolic and regulatory networks. Finally, in literature, tools to repurpose BMCs are described (Lau et al., 2018; Lee et al., 2019). For all these reasons, compartmentalizing the methanol assimilatory pathways seems to be an approach worthy of consideration to establish synthetic methylotrophy in prokaryotes.

Changing a lifestyle from heterotrophic to methylotrophic represents major engineering that have been partially achieved. In 4–10 years from now, it is likely that the next generation of synthetic methylotrophs will be further developed for the production of chemicals (e.g. bulk chemicals, fuels) from methanol, after which commercial production may be feasible. However, there is undoubtedly a long way ahead to achieve a synthetic strain able to use methanol for the production of both biomass and chemicals.
